# Short-Term Choir Singing Supports Speech-in-Noise Perception and Neural Pitch Strength in Older Adults With Age-Related Hearing Loss

**DOI:** 10.3389/fnins.2019.01153

**Published:** 2019-11-28

**Authors:** Ella Dubinsky, Emily A. Wood, Gabriel Nespoli, Frank A. Russo

**Affiliations:** ^1^Department of Psychology, Ryerson University, Toronto, ON, Canada; ^2^Toronto Rehabilitation Institute, Toronto, ON, Canada

**Keywords:** aging, musical training, speech-in-noise, frequency, hearing

## Abstract

Prior studies have demonstrated musicianship enhancements of various aspects of auditory and cognitive processing in older adults, but musical training has rarely been examined as an intervention for mitigating age-related declines in these abilities. The current study investigates whether 10 weeks of choir participation can improve aspects of auditory processing in older adults, particularly speech-in-noise (SIN) perception. A choir-singing group and an age- and audiometrically-matched do-nothing control group underwent pre- and post-testing over a 10-week period. Linear mixed effects modeling in a regression analysis showed that choir participants demonstrated improvements in speech-in-noise perception, pitch discrimination ability, and the strength of the neural representation of speech fundamental frequency. Choir participants’ gains in SIN perception were mediated by improvements in pitch discrimination, which was in turn predicted by the strength of the neural representation of speech stimuli (FFR), suggesting improvements in pitch processing as a possible mechanism for this SIN perceptual improvement. These findings support the hypothesis that short-term choir participation is an effective intervention for mitigating age-related hearing losses.

## Introduction

As the population ages, and the expectation of longevity increases, a growing interest in healthcare is the promotion of *healthy aging* – the maintenance of mental, social, and physical wellbeing as one ages, in order to retain independence and lead a high-quality life. Aging is associated with declines in cognitive functioning (e.g., decreased working memory and attentional control; for review, see Fabiani, 2012), and deteriorating sensory-perceptual processes (e.g., Fozard, 1990). Declines in hearing can make it difficult for aging individuals to maintain personal relationships and engage socially, and have been linked to feelings of isolation and depression (Arlinger, 2003; Djernes, 2006). Although assistive technologies (e.g., hearing aids) can target aspects of peripheral hearing loss, persistent perceptual deficits are widely reported (e.g., Killion, 1997). One prevalent example is the loss of the ability to perceive speech in a noisy environment (Salomon, 1986; Chmiel and Jerger, 1996; Gomez and Madey, 2001; Ricketts and Hornsby, 2005; Betlejewski, 2006). Counseling programs may improve communication outcomes associated with age-related auditory declines, but they do not appear to influence speech-in-noise problems (Hickson et al., 2007, 2019). While some auditory rehabilitation programs have been shown to be moderately effective in mitigating speech-in-noise problems (Kricos and Holmest, 1996; Sweetow and Sabes, 2006; Song et al., 2012), they require a high level of motivation and are not appropriate for all cases (Sabes and Sweetow, 2007; Saunders et al., 2016). As such, there is presently a great demand for complementary interventions that target age-related auditory declines, particularly ones that are engaging and scalable, and that show efficacy with regard to speech-in-noise perception. Developing and evaluating an intervention – and its proposed mechanism(s) for change – involves consideration of biological and experiential contributors to these abilities, beginning with age-related hearing loss and the role it plays in speech-in-noise perception.

Hearing loss can occur at different stages in the auditory system. *Peripheral hearing loss* refers to the reduction in efficient sound transmission through the bones of the middle ear (*conductive hearing loss*), and the deterioration of the outer and inner hair cells (*sensorineural hearing loss*; Arlinger, 2003; Yueh et al., 2003; Wingfield et al., 2005). *Central hearing loss* refers to the degradation of neural mechanisms that relay sound information from the cochlea to the brain, resulting from long-term attenuation of neural input from the cochlea, as well as age-related changes in neuronal responses to sound (Syka, 2002; Frisina and Walton, 2006; Yamasoba et al., 2013). Although peripheral losses can be remediated to some degree through the use of assistive technologies such as hearing aids (or, in extreme cases, cochlear implants), central processing deficits seem to persist in spite of such interventions (Chmiel and Jerger, 1996; Killion, 1997). These central processing deficits – including age-related declines in the synchrony of neural firing (Pichora-Fuller and Schneider, 1992; Frisina and Frisina, 1997; Pichora-Fuller et al., 2007), length of recovery time (Walton et al., 1998), and numbers of neurons in auditory nuclei (Frisina and Walton, 2006) – have been associated with age-related losses in key auditory perceptual abilities, such as sound localization (Abel et al., 2000), pitch discrimination (Raz et al., 1989), duration judgments (Fitzgibbons and Gordon-Salant, 1994; Schneider et al., 1994), mistuned harmonic detection (Alain et al., 2001), and speech-in-noise perception (Pichora-Fuller et al., 1995; Russo and Pichora-Fuller, 2008; Schneider et al., 2010). Of the perceptual deficits, the loss of speech-in-noise perception seems to have the most severe impact on the aging adult’s quality of life (e.g., Pichora-Fuller et al., 1995, 2007; Anderson et al., 2011).

Speech-in-noise perception refers to the ability to track a voice in a complex acoustic environment, such as a crowded room with many people talking. Vital in social settings and everyday interactions, the loss of this skill can immensely impact an individual’s ability to maintain independence, emotional wellbeing, and quality of life as they age (Salomon, 1986; Gomez and Madey, 2001; Betlejewski, 2006). This age-related decline also appears to persist in spite of peripheral remediation, and can even occur in adults with normal audiometric thresholds (Cruickshanks et al., 1998; Schneider B. A. et al., 2002; Tremblay et al., 2003; Gordon-Salant, 2005; Souza et al., 2007; Vermiglio et al., 2012; Alain et al., 2014); in research studies involving older individuals, pure-tone thresholds tend to be a poor predictor of speech-in-noise perception (Dubno et al., 1984; Hargus and Gordon-Salant, 1995; Kim et al., 2006; Souza et al., 2007).

One way to elucidate the neural underpinnings of speech-in-noise perception is through the use of electroencephalography (EEG recordings) to study cortical and subcortical responses to acoustic stimuli (Tremblay et al., 2003; Musacchia et al., 2008; Anderson et al., 2011). Of particular interest here, the *auditory brainstem* – a collection of nuclei involved in afferent and efferent auditory processing – has been shown to encode spectral and temporal acoustic information with a high degree of precision (Clinard et al., 2010; Skoe and Kraus, 2010).

One component of the auditory brainstem response (ABR; Skoe and Kraus, 2010) that has been implicated in perceptual deficits – in particular, speech-in-noise perception – is the *frequency following response* (Johnson et al., 2005; Skoe and Kraus, 2010). This response consists of phase-locked neural activation, wherein the inter-spike intervals correspond to the fundamental frequency (F0) of the sound input (Hoormann et al., 1992). On the basis of animal work involving ablations, the primary source of the FFR appears to be the *inferior colliculus* (Smith et al., 1975), however recent work also suggests cortical contributions (Lehmann and Schönwiesner, 2014; Coffey et al., 2016, 2017a).

The FFR provides a useful index of the auditory nervous system’s representation of periodic sound – such as a vowel in speech – through sustained synchronous neural phase-locking. Spectral and temporal features of the FFR, obtained through signal analysis, are associated with different aspects of neural pitch encoding. A *fast Fourier transform (FFT)* of the signal yields a spectral analysis that can be used to assess the strength of the neural representation of periodic sound input (Skoe and Kraus, 2010). Another feature, *the inter-trial phase coherence (ITPC)*, can be used to assess the extent of consistency in the neural response to periodic sound input – i.e., the extent of phase alignment (synchronization) in oscillatory responses (e.g., Delorme and Makeig, 2004).

In the perception of speech cues, the ability to discern and track changes in pitch over time gets significantly more difficult when the signal-to-noise ratio (SNR) decreases (e.g., Killion et al., 2004). By the time an acoustic signal reaches the auditory cortex of an aging adult, it is likely to have undergone both peripheral and neural distortion (due to age-related declines in sensorineural hearing, and neural noise introduced as the signal is relayed through the ascending auditory pathway, respectively), leading to diminished preservation of key temporal and spectral characteristics (e.g., Yueh et al., 2003; Clinard et al., 2010). This suggests a possible mechanism for age-related declines in speech-in-noise perception (and other auditory perceptual abilities which rely on pitch discrimination), whereby age-related central processing deficits (such as reduced FFR fidelity) result in downstream perceptual impairments which tend to persist in spite of peripheral remediation. In terms of mitigating and preventing these declines, one activity that appears to confer some benefits against certain age-related auditory losses is musical experience (e.g., Alain et al., 2014).

Musicianship is purported to have some benefits outside the musical domain, but the most convincing positive effects have been observed in regards to the auditory system (for review, see Herholz and Zatorre, 2012). Over the course of training, musicians are taught to attend to fine-grained acoustic features – including pitch, timing, and timbre – that contribute to human perception of sound (Kraus et al., 2009; Kraus and Chandrasekaran, 2010). This trained sensitivity to minute acoustic changes is thought to promote the enhancement of auditory perceptual abilities, including those which decline throughout the aging process (Musacchia et al., 2007; Parbery-Clark et al., 2012; Zendel and Alain, 2012; Alain et al., 2014). In studies comparing auditory perception in musicians and non-musicians, musical experience has been associated with a relative advantage in processing some of the same features that have been linked to age-related declines. These benefits include improved pitch discrimination (Kishon-Rabin et al., 2001; Micheyl et al., 2006; Schellenberg and Moreno, 2009; Bidelman et al., 2011b; Meha-Bettison et al., 2018), gap and duration judgments (Rammsayer and Altenmüller, 2006; Zendel and Alain, 2012; Habibi et al., 2014; Donai and Jennings, 2016), mistuned harmonic detection (Koelsch et al., 1999; Zendel and Alain, 2009), and perception of speech-in-noise (Parbery-Clark et al., 2009; for review see Coffey et al., 2017b).

Musicians also demonstrate structural and functional differences in the neural substrates of auditory, sensory-motor, and visuospatial processing (Musacchia et al., 2007; Kraus and Chandrasekaran, 2010; Schlaug, 2015). Among musicians, musical aptitude is correlated with an increase in gray matter volume in the primary auditory cortex, as well as somatosensory and motor areas, the inferior temporal gyrus, hippocampus, and corpus callosum regions (Schlaug et al., 1995; Schneider P. et al., 2002; Gaser and Schlaug, 2003; Herdener et al., 2010). In addition to structural changes in associated brain regions, musicians also demonstrate functional improvements in neural responses to sound, at cortical and subcortical levels in the auditory processing pathway. Compared with non-musicians, musicians demonstrate enhanced neural responses and activation in the auditory cortex (Koelsch et al., 1999; Schneider P. et al., 2002; Pantev et al., 2003; Shahin et al., 2003; Kuriki, 2006; Besson et al., 2007; Zendel et al., 2015; Habibi et al., 2016) and the auditory brainstem (Musacchia et al., 2007; Wong et al., 2007; Lee et al., 2009; Parbery-Clark et al., 2009; Strait et al., 2009; Bidelman and Krishnan, 2010). Notably, musicians demonstrate improvements in both FFR strength (Musacchia et al., 2007, 2008; Bidelman et al., 2011b; Slater et al., 2017) and consistency (Parbery-Clark et al., 2009; Strait et al., 2009; Bidelman et al., 2011a, b; Skoe and Kraus, 2013; Slater et al., 2017); these benefits appear largely resistant to normal age-related declines (Parbery-Clark et al., 2012; White-Schwoch et al., 2013). Because of the importance of pitch processing across auditory perceptual domains, FFR improvements have been suggested as one of the mechanisms through which musicianship enhances auditory perceptual abilities (Kraus and Chandrasekaran, 2010; Bidelman et al., 2011b; Coffey et al., 2017a).

While the aforementioned studies suggest that musical training can improve speech-in-noise perception, they are cross-sectional studies which should preclude causal inferences (Schellenberg, 2019); further, not all studies have found an effect (Ruggles et al., 2014; Boebinger et al., 2015; Madsen et al., 2017, 2019). One way to resolve these inconsistencies is through the evaluation of musical training outcomes in a controlled experimental design (i.e., a longitudinal context), which a handful of studies have sought to do. These studies essentially provided musical training to non-musicians (with the extent and nature of musical training varying between studies), and administered pre- and post-training assessments to determine whether changes occurred in outcomes of interest. In terms of neural changes, musical training has been found to enhance both structure (Hyde et al., 2009) and function (Fujioka et al., 2006; Lappe et al., 2008; Lappe et al., 2011; Habibi et al., 2016) of the auditory cortex in young children and adults who received musical training, compared to those who did not (control participants). Consistency of the FFR has also been found to be enhanced in adolescents following musical training (Tierney et al., 2015). Children who took part in instrumental music training showed improvements in speech-in-noise perception following 2 years of training (Slater et al., 2015), and younger adults who participated in singing training demonstrated improvements in speech-in-noise perception after only 8 days (Jain et al., 2015). Older adults with 6 months of piano training demonstrated improved cortical responses and speech-in-noise perception following training, suggesting that neural and perceptual benefits can be conferred to aging adults in an intervention context (Zendel et al., 2019).

In addition to improvements in auditory processing, musical training has been linked to enhancements in different aspects of cognitive functioning in older adults – including improvements in working memory and executive control processes – in both cross-sectional (Parbery-Clark et al., 2011; Slevc et al., 2016; Grassi et al., 2017; Mansens et al., 2017) and longitudinal studies (Bugos et al., 2007; Särkämö et al., 2014; Biasutti and Mangiacotti, 2018; Fu et al., 2018). Musical experience has also been shown to alter neural structure and function in regions associated with cognition (Schulze et al., 2011; West et al., 2017); improvements in shared neural substrates for music and non-music domains have been suggested as one possible mechanism for transfer from musical training to speech-in-noise perception (e.g., Kraus et al., 2012).

Taken together, cross-sectional and longitudinal findings suggest that musical training may be able to alter brain structure and function; moreover, it appears to have the capacity to promote enhancements in the same auditory abilities that decline as we age (Solé Resano et al., 2010; Hanna-Pladdy and MacKay, 2011; Zendel and Alain, 2012; Alain et al., 2014), suggesting its use as an intervention to mitigate declines in older adults. Of the forms of music making available, singing may be particularly suited to this purpose.

Singing emerges spontaneously in the first months of life (Papoušek, 1996), and appears to be a universal form of expression (Mithen et al., 2006). Although considerable variability in accuracy exists, the vast majority of adults appear to be able to carry a tune (Dalla Bella et al., 2007; Pfordresher and Brown, 2007; Dalla Bella and Berkowska, 2009). Group singing has been shown to lead to improvements in cooperation (Good and Russo, 2016), social and emotional wellbeing (Hillman, 2002; Bailey, 2005; Hays and Minichiello, 2005; Clift and Morrison, 2011; Creech et al., 2013), and physical and creative outcomes (Beck et al., 2000; Clift and Hancox, 2001; Cohen et al., 2006). Some of these benefits may be mediated by changes in hormonal levels that occur during choral singing: after choir practice, choristers demonstrate decreased cortisol (Beck et al., 2000) and enhanced immune system functioning more generally (Kreutz et al., 2004). Singing in a group can also be highly motivating for older adults (Hillman, 2002; Creech et al., 2013), which may promote intervention adherence. This is of particular import when singing is contrasted with existing auditory rehabilitation programs, which tend to be plagued by low compliance rates and high attrition (Sweetow and Sabes, 2010; Tye-Murray et al., 2012).

In addition to the social, cognitive, and emotional benefits, singing appears better positioned to confer *near-transfer* benefits to speech. All forms of vocal production involve the rapid integration of auditory and vocal-motor systems (Hickok, 2001; Zatorre et al., 2007; Pfordresher and Dalla Bella, 2011; Pruitt and Pfordresher, 2015); this integration requires feedback loops along the auditory dorsal stream that allow for real-time monitoring and adjustments (Houde and Jordan, 1998; Brainard and Doupe, 2000; Zheng et al., 2010). In a recent study that compared temporal lobe activations across perception of singing, instrumental music, and speech, it was found that compared with instrumental music, singing and speech both led to greater bilateral activations of the superior temporal sulcus (STS; Whitehead and Armony, 2018), a critical node in the auditory dorsal stream (Hickok et al., 2003).

In terms of perceptual processes, there is greater reliance on the vocal-motor system in more challenging listening environments, such as understanding speech-in-noise (Du et al., 2014); older adults rely on this to an even greater degree (Du et al., 2016). Training the vocal-motor system through singing could theoretically improve the resources upon which older adults draw to perceive degraded speech signals. An emphasis on pitch training, feedback, repetition, and the rewarding nature of improvements have been implicated as key components of successful auditory training paradigms, and in the transfer of musical experience to speech perceptual benefits (Besson et al., 2011; David et al., 2012; Herholz and Zatorre, 2012; Shepard et al., 2013; Patel, 2014; Pruitt and Pfordresher, 2015).

The current study investigated whether short-term choir participation and musical training could improve speech-in-noise perception in older adults, compared to an age- and audiometrically matched control group who were not taking part in musical training. Outcomes of interest included speech-in-noise perception (SIN) and pitch discrimination (FDL), strength and consistency of the neural response to sound (as indexed by features of the frequency following response [FFR] to a repeated speech stimulus), and exploratory cognitive measures of working memory (LSpan) and inhibitory control of attention (Flanker task). We hypothesized that older adults who took part in 10 weeks of group choral practice (2 hours weekly) and individual online musical training (up to 1 hour weekly) would demonstrate improved speech-in-noise perceptual abilities following training, which may be driven in part by enhancements in pitch processing and perception, as indexed by enhanced neural responses to sound (features of the FFR) and improved pitch discrimination thresholds. Exploratory cognitive measures of working memory and attention were assessed in relation to training outcomes, as potential dependent variables. We hypothesized that choir participants would experience greater post-training gains than an age- and audiometrically matched do-nothing control group.

## Materials and Methods

### Participants

**FIGURE 1:**
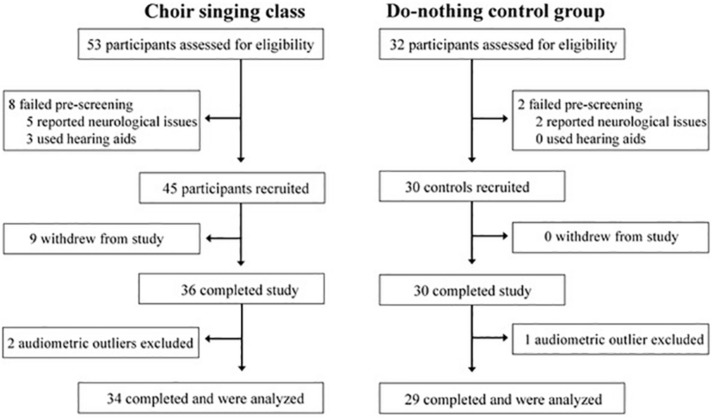
Flow chart showing recruitment and participation of study participants.

The process of recruitment and participation is shown in Figure 1. Participants were recruited from the first class in a 10-week group singing course run through the 50+ Program at Ryerson University; interested participants came into the lab to undergo eligibility testing that week. Fifty three participants were screened (8 didn’t meet eligibility criteria), 45 participants enrolled (9 withdrew from the study), and 36 participants completed choral training and all test sessions. Two participants were rejected as audiometric outliers, so the final analysis included 34 choir singers.

**FIGURE 2:**
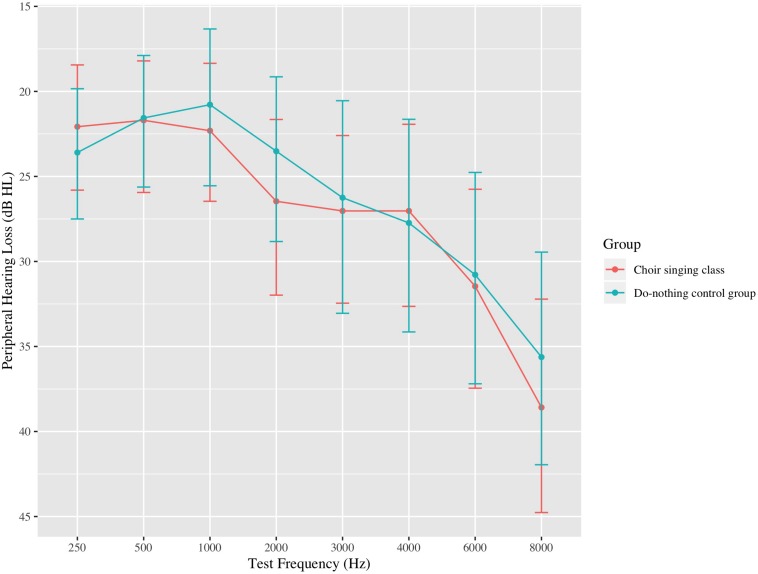
Average bilateral pure-tone thresholds at standard test frequencies up to 8 kHz for participants in the choir singing class and the do-nothing control group. Error bars represent 95% confidence intervals around the means.

Thirty-four choir participants (31 female), aged 54–79 (mean age = 67.6, standard deviation [*SD*] = 6.1 years) underwent pre-testing data collection during the first week of the choir and post-testing data collection following the final choir class. Peripheral hearing loss was measured by an in-lab audiometric assessment at standard test frequencies between 0.25 and 8 kHz; average peripheral hearing loss ranged from 9.7 to 45.3 dB HL (mean = 23.1, *SD* = 9.9 dB HL). Participants were pre-screened to ensure that they did not have any neurological conditions and did not use assistive technology (e.g., hearing aids; see Figure 1). Twenty-nine age- and audiometrically matched *do-nothing control* participants (26 female) aged 60–76 (mean age = 67.7, *SD* = 4.9 years) were recruited through the Ryerson University Hearing Database. Control participants’ average peripheral hearing loss ranged from 10.6 to 47.5 dB HL (mean = 24.1, *SD* = 10.3 dB HL); average pure-tone thresholds for both groups are shown in Figure 2. Groups were matched for the duration of time between test sessions, and there were no group differences in previous musical experience, as indexed by years of formal musical training. Informed consent was obtained from each volunteer prior to their participation in the study, in accordance with the Ryerson Research Ethics Board guidelines (REB 2013-128).

### Study Design

Each choir participant (*n* = 34) visited the lab for a pre-training assessment that took approximately three hours, during which time they completed several questionnaires and auditory and cognitive assessments, and underwent an EEG during presentation of repeated auditory stimuli.

Choir-singing participants took part in weekly 2-hour group choral sessions over the course of 10 weeks, during which time they received pitch training and vocal direction in an open and encouraging environment. In addition to the weekly group choir sessions, participants were assigned weekly individual online musical and vocal training exercises (up to 1 hour weekly). This training consisted of pitch discrimination and vocal production exercises designed to target and improve the participants’ abilities to perceive and produce small changes in pitch (Theta Music Trainer)^1^.

After 10 weeks of choir participation and online musical training, each choir participant returned to the lab for a post-training assessment that lasted approximately 2.5 hours. During this session, participants completed different versions of the original assessments, and underwent a post-training EEG of during auditory stimulus presentation. To account for possible differences in version difficulty within the matched behavioral tasks, participants were assigned one of four possible counterbalanced configurations of assessments.

The *do-nothing control group* (*n* = 29) underwent the same battery of pre- and post-testing, with 8–10 weeks between data collection sessions, but did not receive any active training during this time. The inclusion of this control group in the analysis intended to account for any practice effects within the repeated measures, enabling a controlled examination of the unique effects of the musical intervention on experimental outcomes.

### Experimental Procedure

Apart from the questionnaires, all assessments were completed in an Industrial Acoustics Company (IAC) double-walled sound-attenuating booth. Computerized assessments were presented using a Mac mini (Apple, 2010), with visual components of the experiment presented on a 24′′ Acer LCD display (Acer X243w, 1920 × 1200) placed at eye level approximately 0.5 m in front of the participant. Audiometric testing and FFR auditory stimuli were administered through binaural foam insert headphones (Electro-Medical Instruments, 3A) connected to a GSI 61 Clinical Audiometer (VIASYS Healthcare). All other auditory assessments were administered binaurally through Koss SB40 headphones at approximately 70 dB SPL.

Before the experiment began, participants were familiarized with task requirements and response methods for each assessment. Participants were monitored throughout the data collection session.

#### Questionnaires

After signing the consent form and going over experimental expectations and volunteer rights, participants were given background and music history questionnaires. These elicited demographics and medical history, and years of formal musical training.

#### Auditory Measures

##### Speech-in-noise perception: signal-to-noise ratio (SNR)

Ability to track speech in a noisy environment was assessed using the QuickSIN test (Speech-In-Noise; Etymotic Research; Killion et al., 2004). Participants were presented with four sets of six pre-recorded sentences, with five key words per sentence embedded in four-talker babble noise. In this assessment, the sentences were presented binaurally with a decreasing SNR: the first sentence was presented with an SNR of 25 dB (i.e., the target sentence was twenty-five dB above the background noise; *very easy*), each subsequent sentence was presented with a −5 dB SNR reduction, to an SNR of 0 dB for the final sentence. Participants were asked to repeat back the target sentences as closely to what they heard as possible, and were awarded one point for each correctly repeated target word, for a possible total of 30 points per set. The sentences in the QuickSIN do not contain many semantic or contextual cues, despite being syntactically correct (Wilson et al., 2007). Out of the four sets of sentences presented, the first two lists were treated as practice sets, to familiarize participants with the task requirements, and the second two lists were scored as experimental data. Mean SNR loss (dB) for each list was calculated by subtracting the total number of correct words from 25.5; Mean SNR loss (dB) represents the increased SNR required to correctly repeat 50% of key words on the QuickSIN test (Etymotic), above 2 dB SNR (the level required for normal hearing individuals to achieve 50% test accuracy; Killion, 1997; Killion et al., 2004). Final scores were calculated by averaging the scores of the two experimental lists; since this is a threshold assessment, a more negative SNR score indicates better performance. Participants’ responses were scored online by a researcher, and were also recorded using Audacity software in case response ambiguity necessitated further review. The pre- and post-testing lists consisted of different sentence sets in order to avoid practice effects, and participants’ exposure to the sets were counterbalanced across experimental conditions.

##### Pitch discrimination: frequency difference limens (FDL)

Participants’ ability to distinguish different frequencies was measured using a computerized assessment of FDL. In this task, participants were presented with 3 pure tones, each lasting 200 ms, with amplitude envelopes of 20 ms rise and delay times. A three-alternative forced choice paradigm was used, in which each presented set contained two pure tones at the standard 500 Hz frequency, and one stimulus at a randomly selected higher frequency (Schneider, 1997; Parbery-Clark et al., 2009; Russo et al., 2012). The participant was instructed to identify which tone was higher than the other two by pressing the corresponding number on a computer keyboard (i.e., 1 = first tone is higher; 2 = second tone is higher; 3 = third tone is higher). An adaptive staircase procedure was used to determine the pitch discrimination threshold, whereby the difference between standard and comparison frequencies was halved after three correct responses, or doubled after one incorrect response. After five reversals, the step was changed, so that the frequency difference was divided by 1.414 after 3 correct responses or multiplied by 1.414 after one incorrect response. FDL was determined from the mean of the last 10 reversals.

#### EEG Measure: The Frequency Following Response (FFR)

##### Stimulus

Auditory presentation of a repeated/dα/syllable (F0 = 100 Hz) was used to elicit the FFR, following methodological conventions described by Skoe and Kraus (2010). This stimulus was selected because it is a speech sound that has been extensively used in this area of research, and robustly elicits clear FFRs (Russo et al., 2005; Parbery-Clark et al., 2009, 2012; Skoe and Kraus, 2010). Each participant heard 6000 repetitions of this 170 ms sound, presented at alternating polarities. Stimuli were presented binaurally through insert headphones; stimulus volume was set to 60 dB SPL for normal hearers. For individuals with hearing loss above 25 dB, presentation volume was set to 60 dB + (dB HL – 25 dB), controlling stimulus levels for sensory loss across all participants.

##### EEG administration and data collection

EEG data were collected using a vertical one-channel montage configuration, using three electrodes, in which active and reference electrodes were placed on the mastoids, and a ground electrode was placed on the forehead. A researcher applied 1′′ square cloth solid gel electrodes (EL504, BIOPAC Systems, Inc.) to the mastoids and forehead; electrodes were connected to a BIOPAC MP150 data acquisition system and ERS100C Evoked Response Amplifier (BIOPAC Systems, Inc.). Data were recorded at a sampling rate of 20 kHz, with an online low-pass filter of 10 kHz and a high-pass filter of 1 Hz; the signal was recorded using Acknowledge software (AcqKnowledge, version 4.1). Stimuli were presented for 25 minutes in total, during which time participants shown a silent film^2^, to promote relaxation and stillness during the EEG.

##### EEG data processing

EEG data were processed in MATLAB, using the PHZLAB toolbox (Nespoli, 2016). A 75 Hz high-pass filter was applied, and data were segmented according to individual stimulus responses (i.e., 6000 segments), with epoch windows extending 40 ms pre- and post-stimulus, and the steady-state component extending from 60 to 170 ms post-stimulus onset (Skoe and Kraus, 2010). The 40 ms signal preceding stimulus onset was used as a baseline of ambient EEG activity, against which to compare the response activation. Peak amplitudes in the response waveform were compared to the baseline; response peaks with absolute amplitudes that did not exceed the baseline were not considered ‘reliable’ (Skoe and Kraus, 2010). Myogenic artifacts, which are many times larger than the neural response, were accounted for by rejecting all trials with amplitudes that exceeded a threshold of 50 μV. Responses that remained after artifact rejection were averaged, using the addition method of inverse polarity processing in order to preserve the representation of the fundamental frequency while minimizing stimulus artifact in the signal. Peak amplitude of the fundamental frequency (a measure of the strength of the pitch representation in the signal, or *FFR strength*) was calculated by applying a FFT to the averaged signal, and inter-trial phase coherence (ITPC; a measure of response consistency or *FFR consistency*) was calculated by finding the latency variations across each participant’s un-averaged signal.

#### Exploratory Cognitive Measures

Cognitive assessments were administered electronically on the stimulus computer (see section Experimental Procedure). Assessment scripts, coded in HTML-5, were retrieved from the Millisecond online database^3^ (2016), adapted to have fewer blocks and runtime, and administered using Inquisit software (version 5.0.6). Working memory was assessed using a computerized version of the listening span task (LSpan), an auditory adaptation of the reading span task developed by Daneman and Carpenter (1980). Inhibitory control of attention was assessed using a computerized version of an adapted Flanker task (Ridderinkhof et al., 1997).

### Statistical Analyses

Linear mixed effects analyses in a regression format were conducted on choir and control groups to examine the effects of choir participation on speech-in-noise perception (mean SNR loss; dB), pitch discrimination ability (FDL; Hz), and aspects of the FFR which represent the strength and consistency with which the speech fundamental frequency was represented. Exploratory cognitive measures of auditory working memory (LSpan) and inhibitory control of attention (Flanker effect) were also examined in the same format. In all models, measures were regressed on Session, Group, and the interaction between them (e.g., SNR ∼ Session × Group); contrasts were assigned such that the interaction effect represented the training effect of the choir group compared to the control group. Intercepts significantly varied across participants for all auditory measures; because the Session × Group interaction was the main effect of interest for each outcome measure, individual variability in baseline scores across all dependent variables were included as random effects in the multilevel models. Session × Group interactions are reported first in each section, and significant Session × Group interactions were plotted and broken down in separate multilevel models of the choir and control groups. In these separate regressions conducted on each group, the models specified were the same as the main model for each variable, but excluded the main effect and interaction term involving Group. This was done in order to elucidate differential effects of choir participation vs. do-nothing control participation, on all outcomes of interest. Statistical analyses were conducted in R; the *nlme* and *lmer* packages were used to conduct linear mixed effects modeling in a regression format (Bates et al., 2015; Pinheiro et al., 2019).

## Results

Linear mixed effects models for key auditory measures are summarized in Table 1; pre- and post-testing group means, *post hoc* pairwise *t*-tests, and effect size calculations are reported in Table 2. Due to a computer error, FDL scores for 25 participants were spurious and removed from analyses.

**TABLE 1:** Summary of linear mixed effects models for choir-singing and do-nothing control groups across key auditory measures.

**Mean SNR Loss (dB)**									
	**Overall model**			**Choir-singing class**			**Do-nothing control group**		
**Predictors**	**Estimates**	**95% CI**	***p*-value**	**Estimates**	**95% CI**	***p*-value**	**Estimates**	**95% CI**	***p*-value**
(Intercept)	2.95	[2.15, 3.74]	**<0.001**	3.04	[2.32, 3.76]	**<0.001**	2.95	[2.14, 3.76]	**<0.001**
Session	0.45	[−0.24, 1.14]	0.205	−0.81	[−1.39, −0.23]	**0.006**	0.45	[−0.31, 1.21]	0.247
Group	0.10	[−0.98, 1.18]	0.862						
Session × Group	−1.26	[−2.20, −0.31]	**0.009**						
**Random Effects**									
σ^2^	1.81			1.50			2.18		
τ_00 Participant_	2.95			3.09			2.79		
ICC _Participant_	0.62			0.67			0.56		
Observations	126			68			58		
Marginal *R*^2^/Conditional *R*^2^	0.037/0.634			0.035/0.684			0.010/0.566		

**Pitch Discrimination Thresholds (Frequency Difference Limens; FDL log Hz)**									

(Intercept)	0.94	[0.84, 1.04]	**<0.001**	0.90	[0.81, 1.00]	**<0.001**	0.94	[0.84, 1.03]	**0.001**
Session	−0.03	[−0.10, 0.04]	0.455	−0.13	[−0.21, −0.03]	**0.001**	−0.03	[−0.08, 0.03]	0.322
Group	−0.03	[−0.17, 0.10]	0.623						
Session × Group	−0.11	[−0.20, −0.01]	**0.036**						
**Random Effects**									
σ^2^	0.01			0.02			0.01		
τ_00 Participant_	0.04			0.04			0.04		
ICC _Participant_	0.77			0.70			0.85		
Observations	86			47			39		
Marginal *R*^2^/Conditional *R*^2^	0.082/0.786			0.070/0.791			0.004/0.853		

**FFR Strength at F0 (100 Hz; μV)**									

(Intercept)	0.0118	[0.0091, 0.0144]	**<0.001**	0.0087	[0.0064, 0.0111]	**<0.001**	0.0118	[0.0091, 0.0145]	**<0.001**
Session	−0.0003	[−0.0034, 0.0027]	0.830	0.0033	[0.0008, 0.0058]	**0.009**	−0.0003	[−0.0035, 0.0028]	0.834
Group	−0.0030	[−0.0066, 0.0005]	0.096						
Session × Group	0.0037	[−0.0003, 0.0076]	0.072						
**Random Effects**									
σ^2^	0.0025			0.0025			0.0029		
τ_00 Participant_	0.0023			0.0023			0.0021		
ICC _Participant_	0.45			0.47			0.43		
Observations	112			64			48		
Marginal *R*^2^/Conditional *R*^2^	0.039/0.472			0.055/0.501			0.001/0.421		

*The Session × Group interaction accounted for significant unique variance in speech-in-noise perception (mean SNR loss; dB) and pitch discrimination (Frequency Difference Limens; FDL, log Hz), and marginally significant variance in the strength of the FFR at F0 (μV). Group analyses showed that the choir-singing class demonstrated significant improvements in SNR, FDL, and FFR strength following training, while the control group showed no changes. Bold values indicate statistical significance (*p* < 0.05).*

**TABLE 2:** Mean scores compared with post hoc pairwise *t*-tests and related effect sizes (Cohen’s *d*) for the choir-singing class (*n* = 34) and do-nothing control group (*n* = 29) at pre- and post-testing sessions, across auditory measures of interest.

		**Pre-testing**		**Post-testing**				
		***M***	***SD***	***M***	***SD***	***t* (df)**	***p***	***Cohen’s d***
Choir-singing class	*Speech-in-noise perception (mean SNR loss; dB)*	3.04	2.24	2.24	2.10	2.68(33)	**0.0113**	0.46
	*Pitch discrimination (FDL; log Hz)*	0.87	0.26	0.77	0.22	3.44(19)	**0.0027**	0.77
	*FFR strength at F0 (μV)*	0.0087	0.0065	0.0122	0.0075	−2.31(29)	**0.0284**	0.42
Do-nothing control group	*Speech-in-noise perception (mean SNR loss; dB)*	2.95	2.27	3.40	2.27	−1.14(28)	0.2651	0.21
	*Pitch discrimination (FDL; log Hz)*	0.92	0.25	0.90	0.21	0.98(17)	0.3402	0.23
	*FFR strength at F0 (μV)*	0.0120	0.0066	0.0117	0.0079	0.15(19)	0.8829	0.03

*Bold values indicate statistical significance (*p* < 0.05).*

### Speech-in-Noise Perception

**FIGURE 3:**
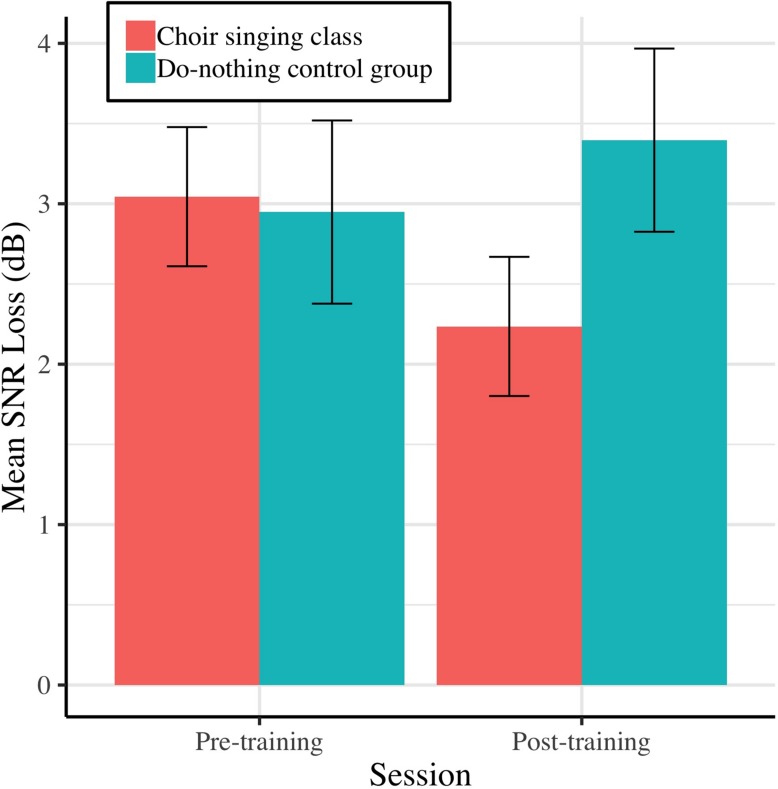
Mean SNR loss (dB) before and after 10 weeks of choir singing or do-nothing control participation, plotted with 95% CIs for repeated measures.

Pre-training and post-training SNR loss (dB) for choir singing and do-nothing control groups is plotted in Figure 3. The Session × Group interaction accounted for significant variance in dB SNR loss [*b* = −1.26, *t*(61) = −2.57, *p* = 0.009]. Regressions conducted on each group showed that choir participants demonstrated improvements of 0.81 dB SNR following training [*b* = −0.81, *t*(33) = −2.68, *p* = 0.006], while control participants showed no change [*b* = 0.45, *t*(28) = 1.14, *p* = 0.247; see Table 1].

### Pitch Discrimination and Neural Representation of Frequency

**FIGURE 4:**
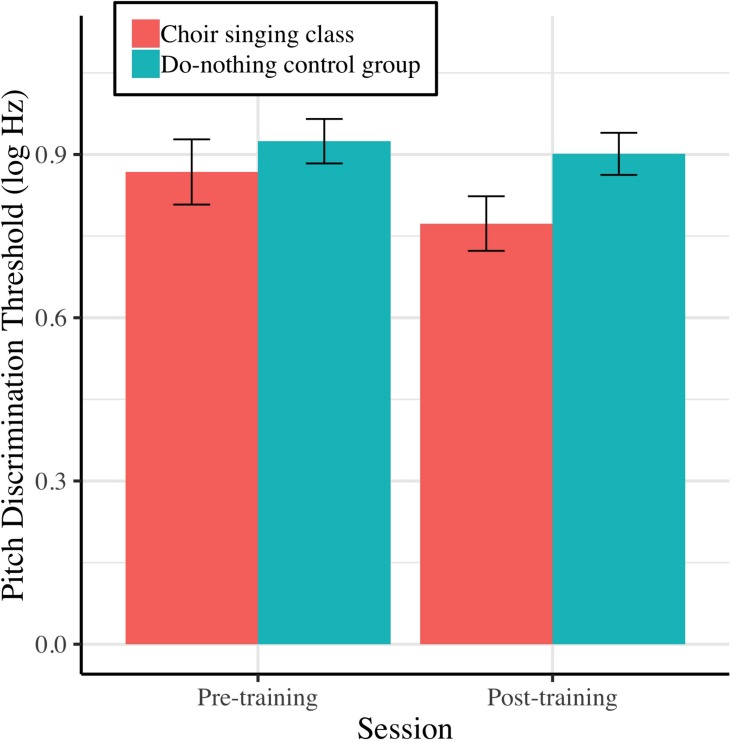
Older adults’ average pitch discrimination thresholds (frequency difference limens; FDL, log Hz) before and after 10 weeks of choir singing or do-nothing control participation, plotted with 95% CIs for repeated measures.

Figure 4 shows mean pitch discrimination thresholds (FDL, log Hz) before and after 10 weeks of choir singing and do-nothing control participation. The Session × Group interaction accounted for significant variance in frequency discrimination thresholds [log Hz; *b* = −0.11, *t*(36) = −2.10, *p* = 0.036]; regressions conducted on each group showed that choir participants demonstrated improved pitch discrimination thresholds following training [*b* = −0.13, *t*(19) = −3.21, *p* = 0.0013], while control participants showed no changes [*b* = −0.03, *t*(17) = −0.99, *p* = 0.322; see Table 1].

**FIGURE 5:**
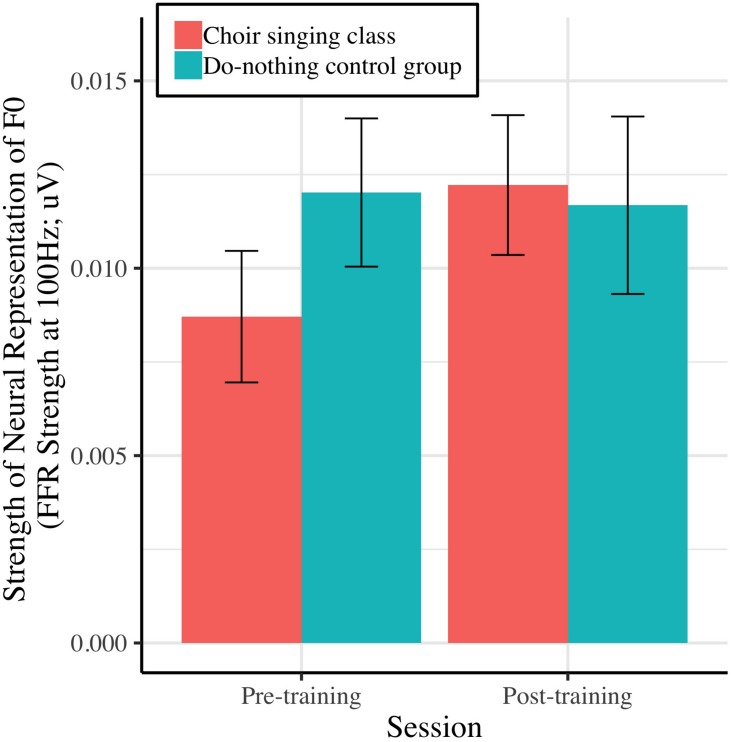
Strength of neural representation (μV) of the fundamental frequency of a speech stimulus (/da/; F0 = 100 Hz), as indexed by the spectral power of the fundamental in participants’ EEG signals (FFR strength at F0).

Figure 5 shows the strength of the neural representation of the fundamental frequency (F0) of the steady-state component of a complex sound (/da/; F0 = 100 Hz), before and after choir-singing or do-nothing control participation. The Session × Group interaction accounted for marginally significant variance in the FFR strength at F0 [μV; *b* = 0.0037, *t*(48) = 1.77, *p* = 0.0721]; regressions conducted on each group showed that following training, choir participants demonstrated improvements in the neural representation of pitch [*b* = 0.0033, *t*(29) = 2.60, *p* = 0.009], while control participants did not demonstrate significant changes [*b* = −0.00034, *t*(19) = −0.21, *p* = 0.8346; see Table 1]. The Session × Group interaction did not account for significant variance in the inter-trial phase coherence of the FFR [μV; *b* = 0.0025, *t*(50) = 0.38, *p* = 0.7066].

### Exploratory Cognitive Measures

There were no Session × Group interaction effects on either listening span (auditory working memory) or the Flanker effect (inhibitory control of attention).

### Possible Contributors to Choir-Driven Improvements in Speech-in-Noise Perception

**FIGURE 6:**
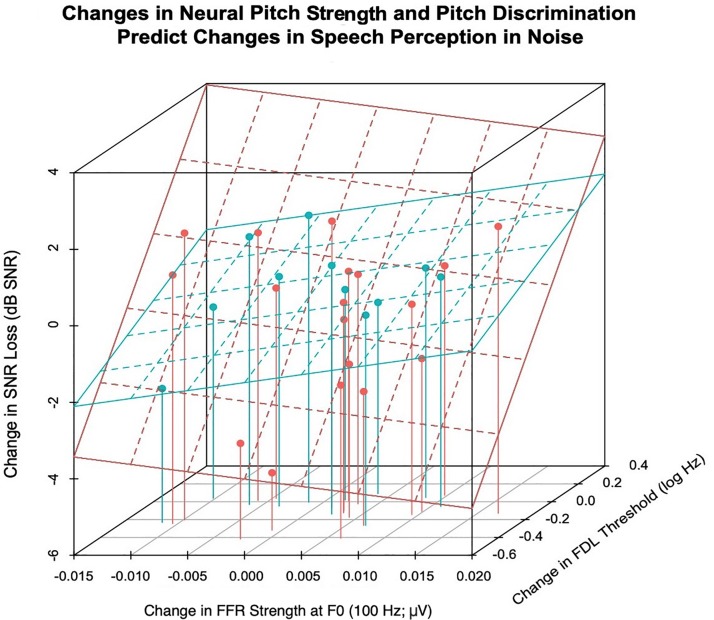
The relationship between improvements in speech-in-noise perception (Δ mean SNR loss; dB), pitch discrimination thresholds (Δ FDL; log Hz), and the strength of the neural representation of frequency (Δ FFR strength at 100 Hz; μV). Choir participants (red) who experienced greater improvements in the neural representation of F0 (more positive FFR strength) and pitch discrimination thresholds (more negative FDL) demonstrated greater improvements in speech-in-noise perception, as indexed by reduced SNR Loss (dB) following training. This relationship was non-significant for the control participants (blue).

Figure 6 shows the relationship between improvements in pitch discrimination ability, FFR strength at F0, and speech-in-noise perceptual gains. SNR scores of choir participants were analyzed in a multilevel model including Session (effect of training), FDL, and FFR strength as potential predictors of variance, in order to elucidate potential mechanisms for choir-related improvements in speech-in-noise perception. Predictors were added into the model hierarchically based on hypothesized contributions to speech-in-noise perception derived from previous research, and only predictors with significant Session × Group interactions were included in the model (i.e., FDL and FFR strength at F0). The final choir model is reported in Table 3; with Session included in the model, the interaction between FDL and FFR strength accounted significant unique variance in SNR scores [*b* = −239.74, *t*(21) = −3.18, *p* = 0.0045]; inclusion of FDL accounted for training-related variance in SNR loss previously accounted for by Session, suggesting a potential mediating effect of pitch discrimination on training-related improvements in speech-in-noise perception.

**TABLE 3:** Hierarchical regression of possible contributors to speech-in-noise perceptual gains following choir (vs. control group) participation; marginal *R*^2^ = 0.325, conditional *R*^2^ = 0.691.

**Choir SNR**			
**Predictors**	**Estimates**	**CI**	**p**
(Intercept)	2.12	1.47 – 2.77	**<0.001**
Session	−0.22	−0.88 – 0.44	0.514
FDL	2.28	0.27 – 4.29	**0.026**
FFR strength	−102.93	−185.49 – −20.38	**0.015**
FDL × FFR strength	−277.14	−548.41 – −5.86	**0.045**
**Random Effects**			
σ^2^	0.90		
τ_00 Participant_	1.07		
ICC _Participant_	0.54		
Observations	44		
Marginal *R*^2^/Conditional *R*^2^	0.325/0.691		

**Control SNR**			

(Intercept)	2.87	1.82 – 3.91	**<0.001**
Session	0.63	−0.44 – 1.70	0.250
FDL	0.32	−3.38 – 4.02	0.864
FFR strength	−45.95	−190.40 – 98.51	0.533
FDL × FFR strength	164.51	−465.17 – 794.20	0.609
**Random Effects**			
σ^2^	1.98		
τ_00 Participant_	2.62		
ICC _Participant_	0.57		
Observations	32		
Marginal *R*^2^/Conditional *R*^2^	0.032/0.584		

*Continuous predictor variables included in the moderation analysis (FDL and FFR strength at F0) were centered around the grand means for each variable before groups were subsetted. Bold values indicate statistical significance (*p* < 0.05).*

### Pitch Discrimination as a Potential Mechanism for Musicianship Improvements in Speech-in-Noise Perception: A Mediation Analysis

**FIGURE 7:**
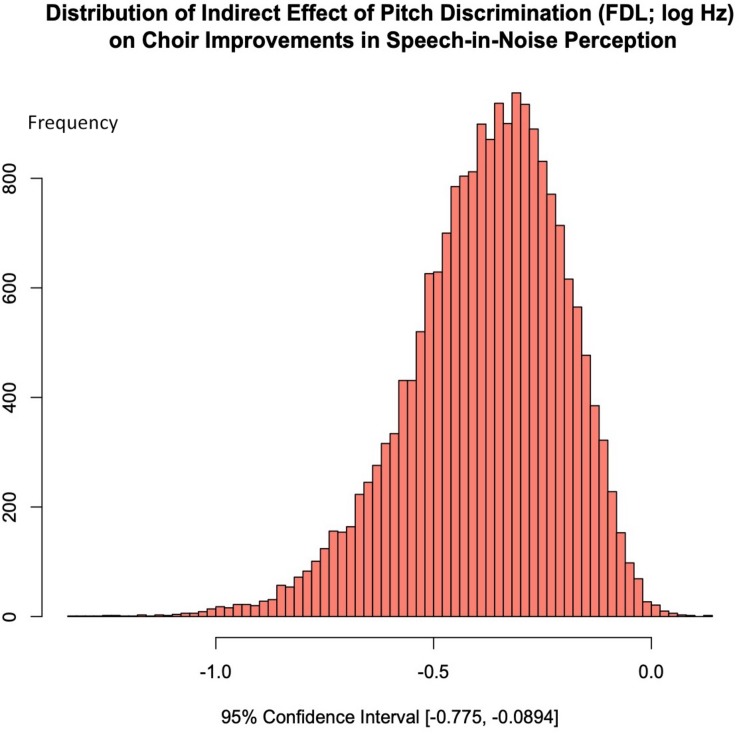
A mediation analyses was conducted using the Monte Carlo technique (20000 samples); choir related gains in SNR were fully mediated by improvements in pitch discrimination ability; 95% CI [–0.775, –0.0894].

Figure 7 shows the distribution of the indirect effect of choir training on SNR, which shows that choir-related improvements in speech-in-noise perception were significantly mediated by improvements in pitch discrimination. The regression of mean SNR loss (dB) on Session, ignoring the mediator, was significant (see Table 1); when mean SNR loss (dB) was regressed on the mediator while controlling for Session, FDL significantly predicted SNR [*b* = 3.57, *t*(18) = 3.22, *p* = 0.0047], but training-specific effect was no longer a significant predictor of SNR [*b* = −0.23, *t*(18) = −0.605, *p* = 0.553]. Aroian’s test revealed a significant mediating effect of FDL on choir-related improvements in SNR (Aroian’s test statistic = −2.20, *p* = 0.0280), and a Monte Carlo resampling approach (*n* = 20000) confirmed that FDL fully mediated the relationship between choir participation and SNR improvements, 95% CI [−0.775, −0.0894]; Figure 7.

**FIGURE 8:**
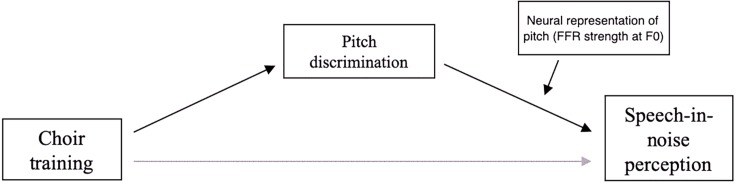
Model of moderated mediation of choir-related improvements in speech-in-noise perception by pitch discrimination and strength of neural representation of pitch.

The significant interaction effect of FDL × FFR strength on SNR (Table 3) suggested a potential moderating effect of FFR on the relationship between pitch discrimination and SNR, so a moderated mediation analysis was conducted to assess the statistical significance of this effect. This analysis revealed a marginally significant moderation of the mediation by changes in FFR strength [*b* = −277.14, *t*(14) = −1.885139, *p* = 0.0803]; the model of this relationship is shown in Figure 8.

Exploratory analyses considered whether the relationship between choir-related improvements in pitch discrimination and speech-in-noise perception could be predicted by the strength of the representation of F0 in the FFR. Simple slopes analyses on low, average, and high FFR strength at F0 (centered variable ± SD) showed that the relationship between pitch discrimination and SNR was strongest in *high FFR conditions* [i.e., when F0 is strongly represented in the FFR; *b* = 4.28, *t*(14) = 3.29, *p* = 0.0054], weaker in *average FFR conditions* [*b* = 2.28, *t*(14) = 1.08, *p* = 0.0546], and non-significant in *low FFR conditions* [*b* = 0.28, *t*(14) = 0.16, *p* = 0.8714], suggesting that when controlling for session, the strength of the FFR at F0 is predictive of the strength of the relationship between pitch discrimination and speech-in-noise perception. These analyses suggest that neural and perceptual pitch processes play a role in speech-in-noise perceptual ability in older adults, and could mechanistically contribute to a potential musicianship advantage in this domain.

### Effects of Peripheral Hearing Loss on Perceptual and Neural Auditory Outcomes

**FIGURE 9:**
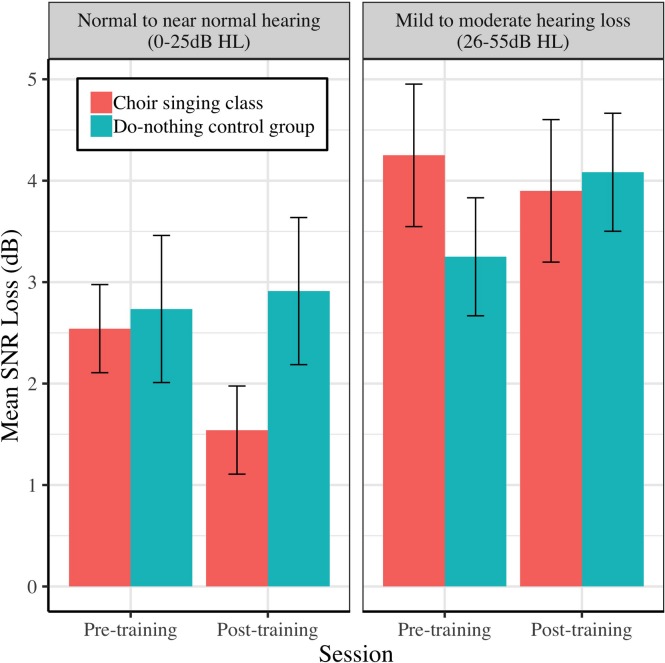
The relationship between peripheral hearing loss and pre-post changes in mean SNR loss (dB), for choir and control groups. Degree of impairment is categorized on clinical audiometric criteria, where normal to near normal hearing = 0–25 dB HL, and mild to moderate = 26–55 dB HL. Error bars are within subjects CIs (95%) plotted around Session × Group means.

**FIGURE 10:**
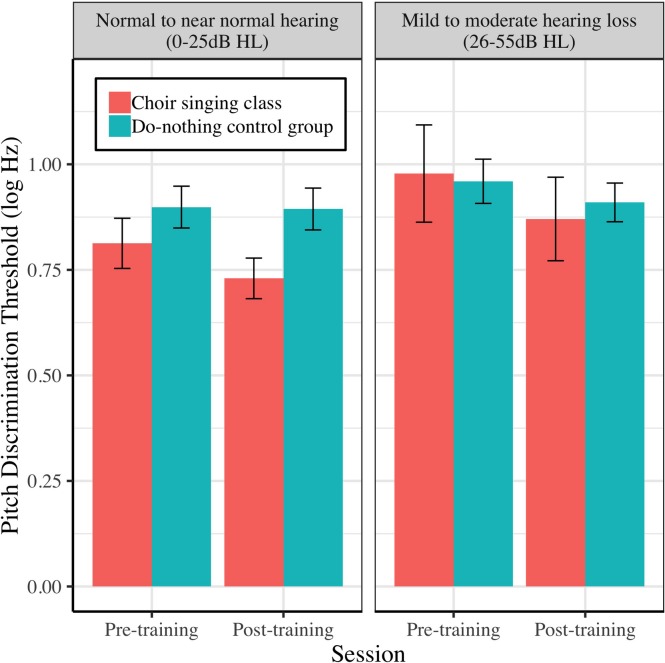
The relationship between peripheral hearing loss and pre-post changes in pitch discrimination thresholds (FDL; log Hz) for choir and control groups. Degree of impairment is categorized on clinical audiometric criteria, where normal to near normal hearing = 0–25 dB HL, and mild to moderate = 26–55 dB HL. Error bars are within subjects CIs (95%) plotted around Session × Group means.

**FIGURE 11:**
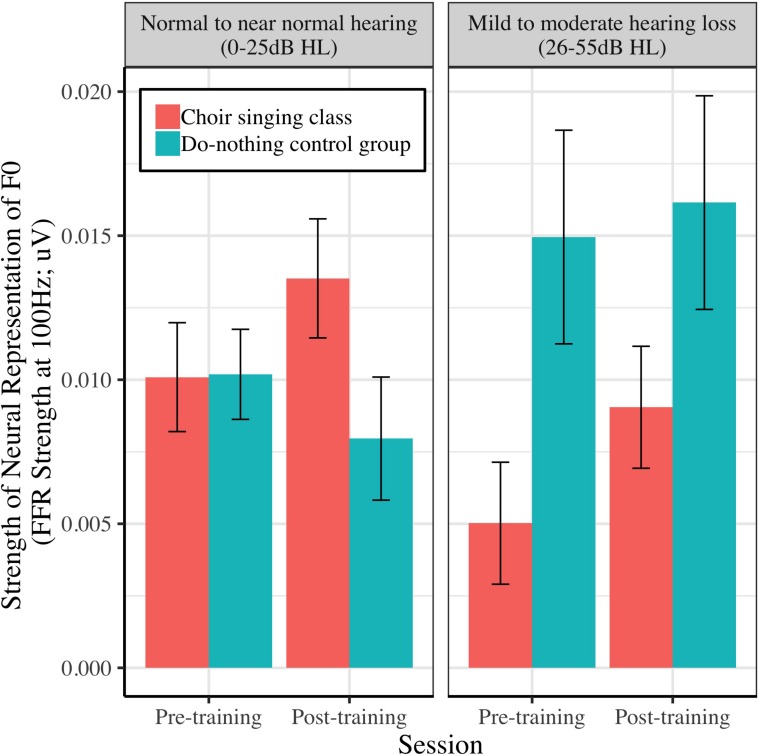
The relationship between peripheral hearing loss and pre-post changes in the strength of neural representation of fundamental frequency (FFR strength at 100 Hz) for choir and control groups. Degree of impairment is categorized on clinical audiometric criteria, where normal to near normal hearing = 0–25 dB HL, and mild to moderate = 26–55 dB HL. Error bars are within subjects CIs (95%) plotted around Session × Group means.

Figures 9–11 demonstrate the differential effects of peripheral hearing loss (dB HL) on the efficacy of the choir-singing intervention on: speech-in-noise perception (Figure 9); pitch discrimination (Figure 10); and FFR strength at F0 (Figure 11). There were significant main effects of Audiometry on SNR [*b* = 0.07, *t*(60) = 2.66, *p* = 0.0101] and FDL [*b* = 0.008, *t*(45) = 2.52, *p* = 0.0153]; across groups and sessions, worse peripheral impairments were predictive of worse performance on perceptual tasks. However, there were no significant effects of peripheral hearing loss on FFR strength at F0, indicating a potential differentiating effect of audiometry on neural vs. perceptual outcomes.

## Discussion

This study demonstrated experimentally that short-term choir participation can be used as an intervention to target and improve speech-in-noise perception in older adults, supporting the hypotheses that: (1) the musicianship advantage in speech-in-noise perception can be conferred to older adults through a relatively short training period, using choir singing and vocal training; and (2) enhancements in pitch processing contribute to improvements in this domain. This study lays the groundwork for a highly scalable, cost-effective, and engaging intervention that can be used to mitigate declines in speech-in-noise perception in older adults, and importantly provides insight into potential neural and perceptual mechanisms underlying these changes. In particular, the relationship between auditory processing, pitch discrimination, and speech-in-noise perception suggested by this study elucidates one way in which musical experience – and specifically, singing and vocal training – can transfer to improvements in speech processing, through enhanced representation of pitch.

Compared with do-nothing control participants, choir singers demonstrated 1.26 dB improvements in mean SNR loss following training, a change that corresponds to a functional difference of 10–20% improvement in speech intelligibility (Middelweerd et al., 1990). Other forms of auditory rehabilitation for older adults yield similar improvements (1.5 dB with LACE training), require intensive practice in the target domain (30 min per day, 5 days per week for 4 weeks), which may account for the relatively poor compliance and high rates of attrition (Sweetow and Sabes, 2006; Song et al., 2012; Tye-Murray et al., 2012). In contrast, group singing is reported to be highly engaging and motivating, provides many benefits outside of the focus of training, and promotes ongoing social involvement and activity (Hillman, 2002; Creech et al., 2013). It is important to note that in the current study, while nine participants withdrew from data collection, almost all of the original 53 participants surveyed remained in the choir class (two withdrew due to health issues), and many participants reported joining other choirs and singing groups after the study ended. As a proof of concept, this makes a strong case for the engagement and enjoyment of participants in a group singing class, and the sustainability of this type of intervention, along with its efficacy at improving speech-in-noise perception.

In terms of possible mechanisms accounting for changes in speech-in-noise perception, improvements appeared to be driven at least in part by enhancements in pitch processing. In addition to improved speech-in-noise perceptual abilities, choir singers demonstrated improved pitch discrimination thresholds (as indexed by lower FDL) and stronger neural representations of the speech fundamental frequency (F0) following training as (stronger FFR representation of F0 of the/da/stimulus; 100 Hz). Analyses showed that training-related improvements in speech-in-noise perception were fully mediated by improvements in pitch discrimination, suggesting that the benefits afforded by choir-singing arose at least in part from enhancements in the perception of pitch. A moderated mediation analysis suggested that over the course of choir training, the strength of the neural representation of F0 was predictive of the strength of the relationship between pitch discrimination and speech-in-noise perception. This suggests that neural indices of pitch processing influence the extent to which older adults rely on pitch cues to support speech-in-noise perception. Taken together, these findings suggest that older adults who take part in 10 weeks of choir singing and vocal training demonstrate enhanced neural responses to and perception of subtle frequency cues, which lead to improved perception of speech-in-noise following training.

A number of previous studies have findings that converge with our mediation account of speech-in-noise via pitch perception. For example, musical experience has been correlated with improvements in speech-in-noise perception (Parbery-Clark et al., 2009; Zendel and Alain, 2012; Swaminathan et al., 2015; for review see Coffey et al., 2017a), pitch discrimination ability (Micheyl et al., 2006; Parbery-Clark et al., 2009; Schellenberg and Moreno, 2009; Bidelman et al., 2011a, b; Fuller et al., 2014; Boebinger et al., 2015; Yates et al., 2018), and subcortical encoding of F0 (Parbery-Clark et al., 2009, 2011; Bidelman et al., 2011a, b; Musacchia et al., 2017); relationships have also been demonstrated between pitch perception and subcortical encoding of F0 (Carcagno and Plack, 2011; Coffey et al., 2016; Bianchi et al., 2017), as well as between speech-in-noise perception and pitch processing (e.g., Coffey et al., 2017a). On the other hand, a number of correlational studies have not been able to replicate the musicianship advantage for speech-in-noise perception (e.g., Ruggles et al., 2014; Madsen et al., 2017). Some of this discrepancy may be based on methodological or sampling differences across studies. More generally, limited experimental work in this field has left the nature of these relationships somewhat uncertain (excepting some recent work by Zendel et al., 2019). Zendel et al. (2019) found that older adults (non-musicians) who took part in 6 months of piano training showed improvements in speech-in-noise perception compared with control and video game intervention groups showing no improvements in this domain. Importantly, these individuals were randomly assigned to the interventions in this study, lending credence to the use of musical training to support auditory abilities in older adults.

As a musical intervention, choir singing may be uniquely suited to hone pitch perceptual processes, through activation of existing vocal-motor systems, rapid integration of perceptual and productive processes, and shared neural architecture activated by speech and vocal song. Choir singers have the benefit of both intrinsic auditory and sensorimotor feedback, and can harness existing feedback loops between auditory perception and vocal production – which allow humans to monitor and dynamically alter speech – to rapidly alter and hone vocal output, including production of pitch. These integrative feedback loops and fine-tuned changes may allow choir singers to undergo rapid improvements in both productive and perceptual processes, in a short period of time. Singing is also an intuitive and innate form of music-making, and may be learned (and improved upon) more quickly than learning to play an instrument. This innate quality, along with intrinsic auditory and vocal motor feedback loops, and extrinsic feedback (from the choir director and other singers) create the ideal circumstances to quickly and effectively improve pitch processing and downstream perceptual abilities such as speech-in-noise perception.

While the current study found that improvements in pitch processing fully mediated choir enhancements in speech-in-noise perception, this does not preclude the role of other neural, perceptual, and cognitive contributors to this ability. Previous work suggests that musical training also leads to enhancements in attentional processes involved with speech encoding (e.g., Zendel et al., 2019); auditory working memory has also been implicated in this ability (e.g., Kraus et al., 2012). Further experimental research is necessary to determine the unique contributions of various auditory, cognitive, and neural processes to music-related improvements in speech-in-noise perception in older adults. In addition, the contribution of productive musical training (i.e., singing practice) vs. perceptual training (i.e., learning to listen to differences in pitch) to auditory processing improvements is not clear from the current study. Notably, a recent study found that non-musicians who received targeted pitch perceptual training achieved pitch discrimination thresholds comparable to musicians in 4–8 hours (Micheyl et al., 2006); it is unknown whether these pitch improvements would be sustained over time, or transfer to speech-in-noise perceptual benefits; it is also unclear whether the mechanism by which pitch discrimination is improved – i.e., through targeted psychoacoustic training, vs. through a more naturalistic singing or music listening paradigm – would alter the degree to which pitch perception mediates speech perceptual processes. This further underscores the need for targeted experimental study of musical (and non-musical) perceptual and productive training on auditory abilities, to elucidate the roles and contributions of each. It is also unclear from the current study whether the auditory benefits of choir participation would persist after cessation of training, and whether these benefits would accumulate with additional/long-term choir or musical involvement. These are rich avenues for future research projects, especially in an experimental/longitudinal context.

In addition to the role of pitch, the degree of peripheral hearing loss appeared to influence the amount of gains choir participants experienced as a function of training, whereby participants with lower levels of peripheral hearing loss appeared to experience greater training-related improvements in speech-in-noise perception. This could be suggestive of a possible limit on the efficacy of this intervention at improving perceptual processes in individuals with levels of hearing loss approaching the need for peripheral assistance (i.e., 26–55 dB HL). One potential explanation is that these individuals may not have been able to hear well enough in the classes to pitch-match with other voices, and thus did not receive equivalent experiential benefits from this activity.

Another explanation is that greater peripheral impairments may have led to more substantial central deficits that were recalcitrant to a behavioral intervention in this capacity, at least in the current dose of 10 weeks of choir singing (2 hours/week of group singing, plus 1 hour/week of individual online exercises). However, there were no effects of peripheral hearing loss on the strength of FFR responses, as individuals within the upper range of peripheral hearing loss still showed improvements in the FFR representation of F0. This suggests that while individuals with greater peripheral hearing loss may not receive perceptual benefits from 10 weeks of choir participation, their neural responses may still be enhanced through this experience. An interesting line of inquiry for a future study would be to address whether individuals with greater hearing loss may be able to obtain similar benefits by participating in choir training in conjunction with the use of hearing aids.

Overall, group choral singing appears to be uniquely well suited for this training paradigm, as it encourages singers to produce (and hopefully, perceive) fine-tuned adjustments in pitch structure, which seem to play a major role in improving speech-in-noise perceptual outcomes in this population. The intrinsic relationship with speech, rapid sensory-motor integration, instantaneous feedback afforded by vocal production and auditory perception, and the innate nature of this ability suggest singing as an ideal candidate for improving a speech-related perceptual issue. Group singing is highly motivating, social, and emotionally fulfilling; this is of immense import in developing interventions that will encourage engagement and promote active involvement, especially with older adults. Overall, running a choir is an immensely scalable intervention, requiring minimal cost and equipment (and which could be implemented anywhere), and this study demonstrated that training-related improvements in auditory perception can appear after a very short intervention period (10 weeks of choir singing). The efficacy of this intervention can easily be assessed through experimental manipulations of dose or duration (e.g., using longer periods of choir singing, or assessing persistence of effects post-training), different study populations (e.g., hearing aid users vs. unaided individuals), and with different emphases during the class (e.g., focusing on pitch matching/singing in unison vs. attending to different melodic or harmonic lines). The ease of implementation and scalability of the choir singing paradigm, efficacy at improving auditory abilities in aging adults, and rich opportunity for further investigation suggest choir singing as an ideal framework for examining musical training as an auditory rehabilitation for aging adults.

## Conclusion

Group singing is an intuitive, engaging, and motivating form of music making, that has in previous studies been shown to contribute to social, emotional, cognitive, and physical well-being. The current findings suggest that choir singing can be used as an effective intervention to mitigate age-related losses in auditory perceptual abilities, in as short a time as 10 weeks. Importantly, these findings showed that this intervention improved older adults’ abilities to perceive speech in noisy environments, a key concern in promoting healthy aging. This work provides an empirical basis for a highly scalable and effective intervention that could significantly improve quality of life in older adults.

## Data Availability Statement

The datasets generated for this study are available on request to the corresponding author.

## Ethics Statement

The studies involving human participants were reviewed and approved by Ryerson University Ethics Board (REB). The patients/participants provided their written informed consent to participate in this study.

## Author Contributions

ED and FR conceived and designed the study. ED recruited the participants, collected the data, analyzed the results, and wrote up findings as Master’s thesis with FR as supervisor. EW collected the data, revised the literature review, and contributed to the theoretical interpretation of findings. GN developed MATLAB toolbox for processing EEG data. All authors were involved in discussions about the interpretation of the results and the writing of the manuscript.

## Conflict of Interest

The authors declare that the research was conducted in the absence of any commercial or financial relationships that could be construed as a potential conflict of interest.

## Footnotes

1 https://trainer.thetamusic.com

2 http://www.openculture.com/free-silent-films

3 http://www.millisecond.com/download/library/
